# Yinzhihuang injection induces apoptosis and suppresses tumor growth in acute myeloid leukemia cells

**DOI:** 10.1371/journal.pone.0289697

**Published:** 2023-10-10

**Authors:** Zhe Huang, Yunfu Shen, Xianming Fan, Qulian Guo, Wenzhe Ma

**Affiliations:** 1 Department of Pediatrics, The Affiliated Hospital of Southwest Medical University, Sichuan Clinical Research Center for Birth Defects, Luzhou, Sichuan, China; 2 State Key Laboratory of Quality Research in Chinese Medicine, Macau University of Science and Technology, Macau, China; 3 Department of Respiratory and Critical Care Medicine, The Affiliated Hospital of Southwest Medical University, Luzhou, Sichuan, China; kafrelsheikh University, EGYPT

## Abstract

**Background:**

The unmet needs in treating acute myeloid leukemia(AML) promote us to look for more effective and less toxic therapies. In this study, we discovered that Yinzhihuang injection(YZHI), a traditional Chinese patent medicine for hepatitis treatment, suppressed the growth of AML cells.

**Method:**

Anti-proliferative activities of YZHI were measured by CCK-8 assay. Cell cycle arrest was evaluated by PI staining, and apoptosis was evaluated by annexin V/PI staining. To explore the cell cycle arrest and cell death mechanism induced by YZHI, we assessed a series of assays, including measurements of the protein expression and cellular ATP. The anti-tumor activity was further demonstrated in nude mice.

**Results:**

Flow cytometric and biochemical analysis revealed that YZHI caused cell cycle arrest and induced apoptosis in the AML HL-60 cells. Mechanistically, YZHI activated AMPK by promoting phosphorylation of the kinase. The active AMPK negatively regulated the downstream target mTORC1, leading to the inhibition of cell proliferation and induction of apoptosis. Pretreatment with the AMPK inhibitor compound C rescued YZHI induced apoptosis and partially restored cell proliferation of HL-60. Consistent with the data in vitro, YZHI obviously suppressed subcutaneous xenograft growth in nude mice.

**Conclusions:**

In a word, our data suggest that YZHI can be repurposed for the treatment of AML, which is worthy of further clinical evaluation.

## 1. Introduction

Acute myeloid leukemia (AML), accounting for ~20% of childhood and ~80% of adult leukemia, respectively, is a rapidly progressing hematopoietic malignancy [1, 2]. It is characterized by the accumulation of clonal myeloid progenitor cells that cannot differentiate into mature blood cells and is accompanied by multilineage cytopenias [3]. While it is initially responsive to cytarabine and anthracycline chemotherapy in most patients, the long-term outcomes of AML have not improved significantly for over three decades. Several targeted drugs for AML treatment, including the fms-like tyrosine kinase 3 (FLT3) inhibitors, the mutant isocitrate dehydrogenase (mIDH) inhibitors and the B-cell lymphoma-2 (BCL-2) inhibitors, have been approved in recent years [4–7]. However, the 5-year overall survival rate for patients <60 years remains low, ranging from 35% to 40% [8, 9]. Therefore, novel therapies are urgently needed for AML management.

Traditional Chinese Medicine (TCM) use medicinal herbs to treat and relieve human diseases. Yinzhihuang injection (YZHI) is an injectable polyherbal prescription derived from the ancient Chinese medicine formula of Yinchenhao decoction. It comprises four herbal extracts, including *Artemisia*, *Gardenia Fructus*, *Baicalin*, and *Honeysuckle*, and is widely used to treat acute and chronic hepatitis in China [10, 11]. YZHI alleviates jaundice by promoting bile excretion [12] and attenuates intrahepatic cholestasis by regulating farnesoid X receptor and Mrp2/Bsep [13, 14]. By assessing mouse T-cell proliferation and cytokine/chemokine produced by human peripheral blood mononuclear cells, Chen [15] showed that YZHI is a potent inhibitor of T-cell activation. Yao [16] demonstrated that YZH could activate the AMPK/SREBP-1 pathway to ameliorate hepatic steatosis and diet-induced obesity. However, no anti-tumor effect of YZHI has been reported so far.

Here, we found the anti-AML effects of YZHI and investigated its underlying mechanisms. The study suggests that YZHI is a promising therapy for AML patients.

## 2. Materials and methods

### 2.1 Cell lines and culture conditions

Human AML cell lines (HL-60, KG-1), human Acute lymphoblastic leukemia (ALL) cells lines (CEM-C7, Nalm6, SupB15), human breast cancer cell line MDA-MB-231, human colon cancer cell line HCT116, human normal hepatic cell line Lo2, and human myofibroblast stromal cell line WPMY-1 were purchased from the US Model Culture Collection (ATCC, US). Human AML cell line THP-1 was obtained from the Chinese Academy of Sciences Cell Bank. HL-60, KG-1, THP-1, CEM-C7, Nalm6, SupB15, and MDA-MB-231 cell lines were cultured in RPMI-1640 medium (Gibco) supplemented with 10% fetal bovine serum(FBS, Gibco), 1% penicillin(pen) and streptomycin(strep). HCT116, Lo2, and WPMY-1 cell lines were cultured in DMEM medium (Gibco) supplemented with 10% FBS, 1% pen/strep.

### 2.2 Chemicals

Yinzhihuang injection was purchased from Shenwei Pharmaceutical Co. Ltd. (Batch NO 170613D1, National drug approval Z13020772), Z-VAD(OMe)-FMK (Z-VAD, HY-16658, MCE), sulforhodamine B (SRB, S1402, Sigma Aldrich), Compound C (HY-13418A, MCE), CCK-8 kit (Japan Tongren Chemical Research Institute), Annexin V-FITC Apoptosis Detection Kit I (556547, BD Bioscience), PI/RNase A (KeyGen BioTECH), CellTiter-Glo Assay Kit (G7573, Promega), JC-1 staining (M34152, Invitrogen).

### 2.3 Antibodies

Western blot immunoassay was performed with antibodies against rabbit monoclonal anti-p-4E-BP1(Cat.#2855), rabbit monoclonal anti-cleaved caspase-3(Cat.#9664), rabbit monoclonal anti-4E-BP1(Cat.#9644S), rabbit monoclonal anti-cleaved PARP (Cat.#5625), rabbit monoclonal anti-p70S6 kinase(Cat.#2708), rabbit monoclonal anti-Bax(Cat.#2772), rabbit monoclonal anti-p-p70S6 kinase(Cat.#9234), rabbit monoclonal anti-Bcl-2(Cat.#2876), monoclonal anti-AMPKα (Cat.#2532s), rabbit monoclonal anti-cyclin D1(Cat.#2978), rabbit monoclonal anti-p-AMPKα (Cat.#2535s), anti-mouse secondary antibody(Cat.#7076) and anti-rabbit secondary antibody(Cat.#7074). The above antibodies were all purchased from Cell Signaling Technology. Mouse monoclonal anti-β-actin (Cat.#A5441) was purchased from Sigma Aldrich.

### 2.4 Proliferation assay in vitro

Cell proliferation assay was assessed with the CCK-8 kit(Japan Tongren Chemical Research Institute) on leukemia cell lines. HL-60 cells (2×10^4^ cells/well), CEM-C7 cells(1×10^4^ cells/well), THP-1, KG-1, Nalm6, SupB15 cells (1.5×10^4^ cells/well) were seeded in 96-well plates in a volume of 90 μl/well. Then 10 μl medium containing various doses of YZHI were added and incubated for 72 h at 37°C. CCK-8 solution was added at 5 μl/well and incubated for 4 h at 37°C. The absorbency at 450 nm was measured using the SpectraMax 190 microplate reader (Molecular Devices). The relative cell growth rate was calculated with the following equation: Relative Growth (%) = [OD (treated) -OD (blank)]/[OD (control)-OD (blank)] ×100%. The IC_50_ of YZHI was counted by Graph Pad Prism 8.0 software. The effect of YZHI on the viability of HCT116, MDA-MB-231, Lo2, and WPMY-1 cell lines was evaluated through the sulforhodamine B (SRB) assay [17].

### 2.5 Apoptosis assay

HL-60 cells were seeded in 6-well plates at 5×10^5^ cells/well and treated with YZHI of indicated concentrations. After 24 h, the cells were collected in the tube, washed with cold phosphate-buffered saline (PBS) (pH 7.4), then incubated with 5 μl PI and 5 μl FITC Annexin V in 100 μl 1×Binding Buffer at room temperature in the dark for 20 min. Next, the cells were resuspended in 400 μl 1×Binding Buffer. Cellular apoptosis was assessed with Annexin V-FITC Apoptosis Detection Kit I(BD Bioscience). The cells were analyzed by flow cytometer (BD Bioscience) after passed through a filter. The data were analyzed by FlowJo 7.6 software.

### 2.6 Cell cycle analysis

HL-60 were seeded at 5×10^5^ cells/well in 6-well plates and incubated with medium containing YZHI for 24 h. The next day cells were collected in the tube, washed with cold PBS twice. Then 70% ethanol was added in cold PBS to fix cells for 2 h at -20°C. Next, cells were stained with 500 μl PI/RNase A (KeyGen BioTECH) at room temperature for 30 min. The cell cycle was analyzed by flow cytometry (BD Bioscience) and FlowJo 7.6 software.

### 2.7 Western blotting

HL-60 cells were seeded at 1×10^6^ cells/well in 6-well plates, incubated with medium containing YZHI for indicated time point. Cell lysates were prepared with RIPA buffer supplemented with phosphatase inhibitor (Roche) and protease inhibitor cocktail (Roche). The protein concentrations were quantified by BCA protein assay kit (P0012, Beyotime). Protein samples were heated at 95°C for 10 min following diluted in 5 x loading buffer. Then, 30 μg of each sample was fractionated on SDS-PAGE gels, transferred to PVDF membrane (Millipore) and incubated with different primary antibodies overnight at 4°C. Next, HRP-conjugated secondary antibodies(1:10000) were added at room temperature following the membranes were washed with 1×TBST. Proteins were visualized with SuperSignal West Pico Chemiluminescent Substrate or SuperSignal West Dura Extended Duration Substrate (Thermo Scientific).

### 2.8 Cellular ATP measurement

HL-60 cells were plated in 96-well plates at 6×10^4^ cells/well in duplicate, incubated with medium containing YZHI for 24 h. ATP concentration was determined with CellTiter-Glo Assay Kit (G7573, Promega).

### 2.9 Mitochondrial membrane potential(MMP) measurement

MMP was determined by JC-1 staining (M34152, Invitrogen). HL-60 cells were seeded at 5×10^5^ cells/well in 6-well plates, incubated with medium containing YZHI for 24h. The next day cells were collected in the tube, washed with PBS, then stained with JC-1(2 μM final concentration) in warm medium for 20 min at 37°C. Next, Cells were resuspended in 500 μl PBS following washed with PBS twice. The cells in different groups were analyzed by flow cytometer (BD Bioscience) after passed through a filter. The data were analyzed by FlowJo 7.6 software.

### 2.10 Xenograft assay

All mice used in the animal experiments were 4–6 week old female nude mice. 5×10^6^ HL-60 cells were inoculated subcutaneously into both hind limbs of nude mice. We randomly divided the mice into two groups: vehicle (normal saline, ip), YZHI (135.3 mg/kg/d, ip) when tumor volumes reached 100 mm^3^. The therapy lasted for 21 days and the tumor size and body weight were measured every other day. The tumor volumes were calculated following equation: volume = (width^2^ × length)/2 [18]. All animal studies were approved by the Animal Ethical Committee of Affiliated Hospital of Southwest Medical University. Project codes:20211118–030. Euthanasia was performed by delivering carbon dioxide via compressed gas followed by immediate cervical dislocation.

### 2.11 Statistical analyses

All data were shown as mean ± standard deviation (SD). And statistical analyses were performed through Graph Pad Prism 8.0 software. The data were analyzed by one-way analysis of variance (ANOVA) if comparing the differences more than two groups. Student’s *t* test was selected to compare two groups. * represents *p* < 0.05, ** represents *p* < 0.01, *** represents *p* < 0.001, **** represents *p* < 0.0001.

## 3. Results

### 3.1 YZHI inhibits AML cell proliferation

**Fig 1:**
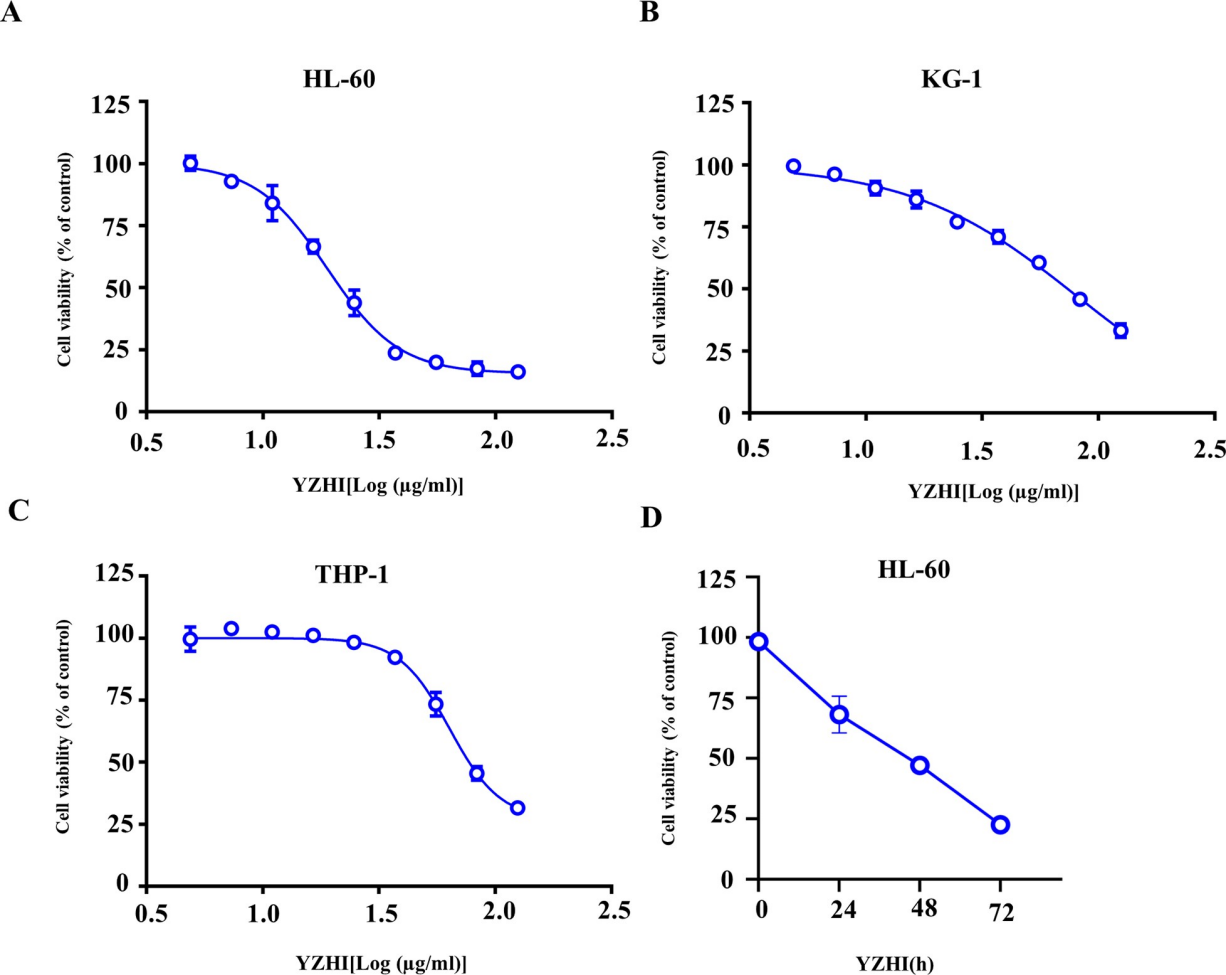
YZHI inhibits AML cell proliferation. (A-C) HL-60, KG-1 and THP-1 cells were treated with different concentrations of YZHI for 72 h. YZHI was first diluted to the highest concentration of 125 μg/ml and then twice diluted in turn. (D) HL-60 cells were treated with 24 μg/ml YZHI for various times. Cell viability (% of control) was performed by CCK-8 assay. (mean ± SD, n =  3 biologically independent samples).

YZHI was first diluted to the highest concentration of 125 μg/ml, corresponding with the maximum clinical doses, to evaluate its cytotoxic effects on cancer cell lines [15]. Then, a serial dilution was prepared and used to incubate the cells. As shown in Fig 1A–1C (S1 Fig), YZHI suppressed the growth of three AML cell lines dose-dependently. The IC_50_ of HL-60, THP-1 and KG-1 were 9.6±2.4 μg/ml, 33.8±5.9 μg/ml and 40.6±7.6 μg/ml, respectively. Furthermore, YZHI did not obviously affect the proliferation of normal cell lines from liver (Lo2) and prostate stroma (WPMY-1), whose IC_50_ were over 125 μg/ml (Table 1, S1 Table). YZHI was also active on ALL cell lines (CEM-C7, Nalm6, SupB15) and breast cancer cell line MDA-MB-231, barely affecting colon cancer cell line HCT116 (Table 1, S1 Table). So, YZHI exhibited a broad anti-cancer spectrum. We focused on AML cells and did the rest of the studies mainly on HL-60, the most sensitive cell line to YZHI treatment. In accord with the dose effect of YZHI, the growth of HL-60 cells was also inhibited in a time-dependent manner (Fig 1D and S1 Fig). The relative cell viability at 24 h, 48 h and 72 h was approximately 68.1%, 47.1%, and 22.6%. These results suggest that YZHI affects the proliferation of AML cell lines in a dose- and time-dependent manner.

**Table 1:** The IC_50_ of YZHI in various cell lines (mean±SD).

Cell line	YZHI IC_50_ for 72h (μg/ml)	Cell line	YZHI IC_50_ for 72h (μg/ml)
**HL-60**	**9.6±2.4**	**CEM-C7**	**26.0±3.0**
**THP-1**	**33.8±5.9**	**SupB15**	**37.6±4.8**
**KG-1**	**40.6±7.6**	**Nalm6**	**17.2±4.3**
**HCT116**	**85.7±4.4**	**Lo2**	**>125**
**MDA-MB-231**	**34.0±4.6**	**WPMY-1**	**>125**

The IC_50_ of YZHI on various cell lines after 72 h treatment.

### 3.2 YZHI induces cell cycle arrest in HL-60 cells

**Fig 2:**
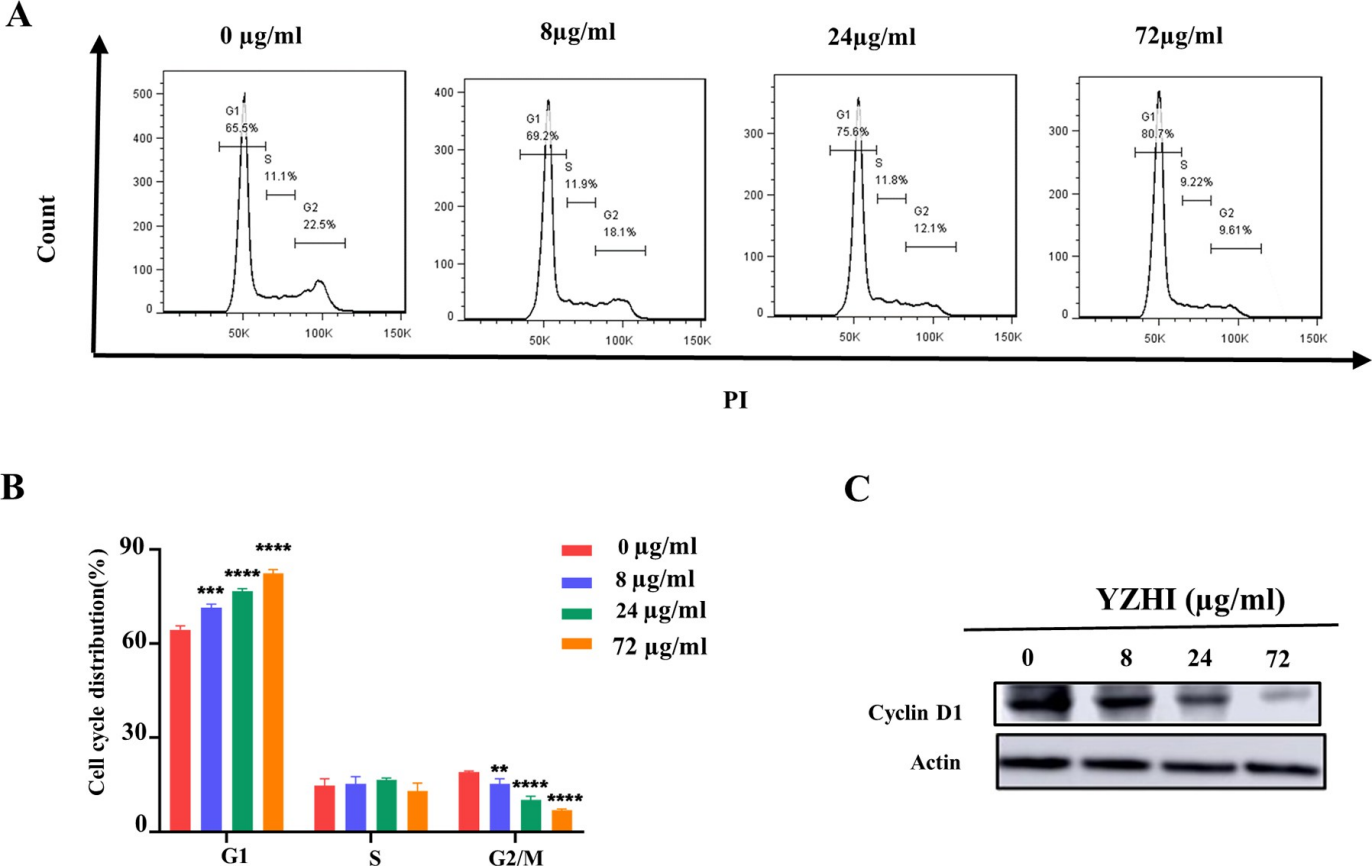
YZHI induces cell cycle arrest in HL-60 cells. (A) Cell cycle analysis of HL-60 cells treated with YZHI. (B) Analyze the total cell population in the three different phases of cell cycle(G1, S and G2/M) after 24 h drug treatment. (C) Western blot analysis of cell cycle marker Cyclin D1 after YZHI treatment for 24 h. All data were analyzed as mean ± SD (n = 3, **p < 0.01, ***p < 0.001, ****p < 0.0001).

We further evaluated the effect of YZHI on cell cycle distribution. Flow cytometry analysis after PI staining revealed that YZHI significantly increased the cell percentage in the G1 phase. In the control group, there was merely 64.39%. But it was increased to 71.45%, 76.75% and 82.4% when YZHI was treated at 8, 24 and 72 μg/ml (Fig 2A, 2B and S2A Fig). Furthermore, in line with causing the cells arrested in the G1 phase, YZHI treatment downregulated the levels of Cyclin D1, an essential regulator of cell cycle progression (Fig 2C and S2B Fig). Taken together, these data suggest that YZHI induces cell cycle arrest in HL-60 cells.

### 3.3 YZHI induces apoptosis in HL-60 cells

**Fig 3:**
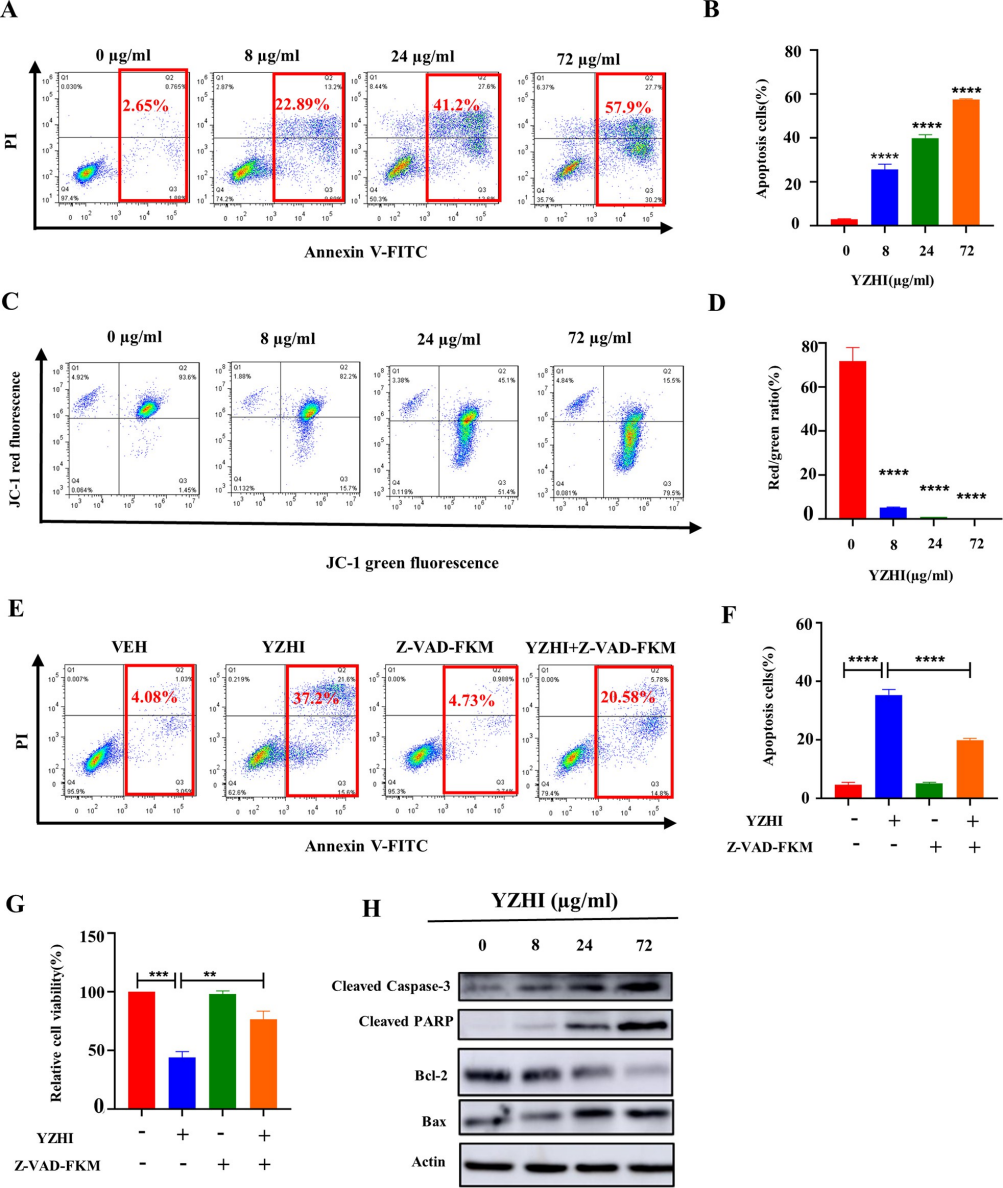
**YZHI significantly induces apoptosis in HL-60 cells.** (A) Flow cytometric analysis of apoptosis in HL-60 cells treated with different concentrations of YZHI. (B) The statistical analysis result of (A). (C) The MMP level of HL-60 cells was assessed after 24 h drug treatment. (D) Statistical analysis of the ratio of Red-green fluorescence intensity. (E) Apoptosis analysis was performed by flow cytometry in HL-60 cells incubated with vehicle or YZHI (72 μg/ml) for 24h following pretreated with vehicle or Z-VAD-FKM (50um) for 2h. (F) The statistical analysis result of (E). (G) Cell viability was determined by CCK-8 assay in HL-60 cells incubated with vehicle or YZHI (8 μg/ml) for 72h following pretreated with vehicle or Z-VAD-FKM (50um) for 2h. (H) Western blot analysis of Bcl-2, Bax, cleaved caspase-3 and Cleaved PARP in HL-60 cells after YZHI treatment. All data were analyzed as mean ± SD (n = 3, **p < 0.01, ***p < 0.001, ****p < 0.0001).

As induction of apoptosis is the most common mechanism of chemotherapeutic drugs on cell proliferation, we next explore whether YZHI can induce apoptosis in HL-60 cells. YZHI significantly increased the percentage of apoptotic cells, as shown in Fig 3A (S3A Fig). In the control group, there was merely 2.65% of apoptotic rate. But it was remarkably increased to 22.89%, 41.2% and 57.9% when YZHI was treated at 8, 24 and 72 μg/ml (Fig 3A, 3B and S3A Fig).

The decrease of MMP is a detection index in the early phase of apoptosis. Therefore, we checked MMP with JC-1 staining and discovered that YZHI significantly induced the reduction of MMP in HL-60 cells (Fig 3C, 3D and S3A Fig). Accordingly, well-known apoptosis markers [19], such as cleaved PARP and cleaved Caspase-3, were induced by YZHI dose-dependently (Fig 3H and S3B Fig). In addition, the antiapoptotic protein Bcl-2 was decreased and the proapoptotic protein Bax was significantly induced with YZHI treatment in HL-60 cells (Fig 3H and S3B Fig). Furthermore, pretreatment with the pan-caspase inhibitor Z-VAD-FMK partially rescued YZHI-induced apoptosis (Fig 3E, 3F and S3A Fig) and, consequently, cell growth (Fig 3G and S3A Fig).

Collectively, we conclude that YZHI induces apoptosis in AML cells from these results.

### 3.4 AMPK/mTORC1 signaling pathway mediates YZHI-induced apoptosis and growth inhibition in HL-60 cells

**Fig 4:**
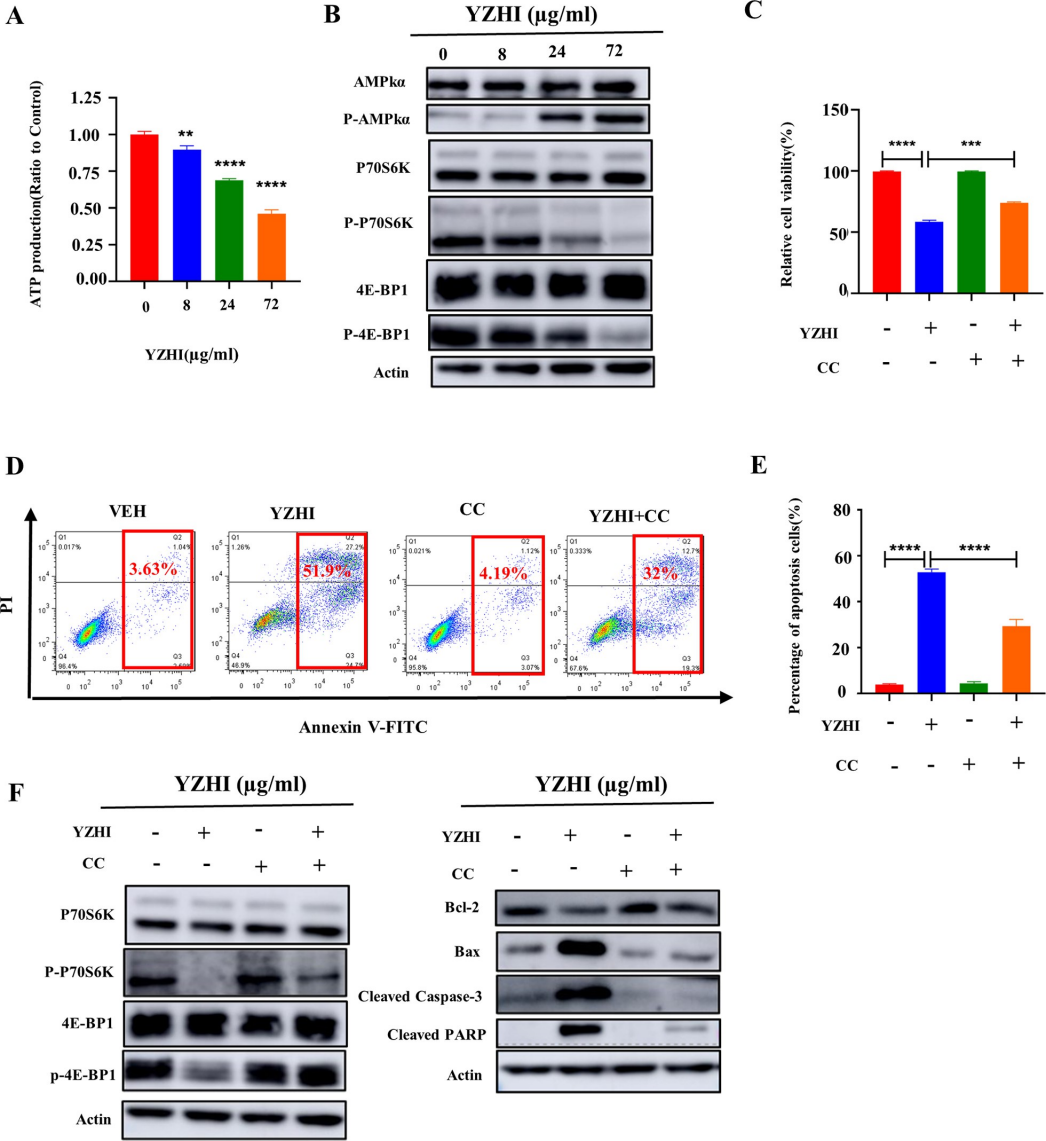
AMPK/mTORC1 signaling pathway mediates YZHI-induced apoptosis and growth inhibition in HL-60 cells. (A) Cellular ATP levels after incubation with YZHI for 24 h. (B) Western blot analysis of proteins in the AMPK/mTORC1 pathway. (C) Cell viability was determined by CCK-8 assay in HL-60 cells incubated with vehicle or YZHI (8 μg/ml) for 72h following pretreated with vehicle or Compound C(5 μm) for 2h. (D) Apoptosis analysis was performed by flow cytometry in HL-60 cells incubated with vehicle or YZHI (72 μg/ml) for 24h following pretreated with vehicle or Compound C (5 μm) for 2h. (E) The statistical analysis result of (D). (F) Western blot analysis of proteins in the cell apoptosis and AMPK/mTORC1 pathway. All data were presented as mean ± SD (n = 3, **p < 0.01, ***p < 0.001, ****p < 0.0001).

To understand the underlining mechanism of YZHI-induced apoptosis, we did network pharmacology-based analysis as previously described [20]. It implied that the 5’ -AMP-activated protein kinase (AMPK) pathway and the cellular energy production might be the priority target of YZHI. To test this possibility, we first measured the intracellular ATP levels in HL-60 cells after YZHI treatment. Indeed, YZHI dose-dependently decreased the ATP levels (Fig 4A and S4A Fig). AMPK is the major intracellular metabolic sensor that helps maintain cellular energy homeostasis and is activated by the phosphorylation of threonine 172 (Thr^172^) upon ATP depletion [21]. As expected, YZHI induced the phosphorylation of AMPK dose-dependently (Fig 4B and S4B Fig).

The mammalian target of rapamycin complex 1 (mTORC1), which controls apoptosis, protein synthesis and cell proliferation [22], is a protein complex and negatively regulated by AMPK [23]. Therefore, we speculated that YZHI induces apoptosis and inhibits cell proliferation through the AMPK/mTORC1 pathway. Indeed, YZHI inhibited p-P70S6K and p-4E-BP1, the effectors of mTORC1 responsive for protein synthesis (Fig 4B and S4B Fig).

To confirm the activation of AMPK is essential for the anti-cancer effect of YZHI, compound C, which is a specific inhibitor of AMPK, is used to block the signaling [24]. We found that compound C partially decreased the apoptosis (Fig 4D, 4E and S4A Fig), reversed the protein levels of p-P70S6K, p-4E-BP1, Bax, Bcl-2, cleaved PARP and cleaved Caspase-3 (Fig 4F and S4B Fig), and rescued cell proliferation of HL-60 cells (Fig 4C and S4A Fig). Therefore, these findings suggest that the AMPK/mTORC1 axis mediates YZHI-induced apoptosis and growth inhibition in HL-60 cells.

### 3.5 YZHI suppresses xenograft growth in vivo model

**Fig 5:**
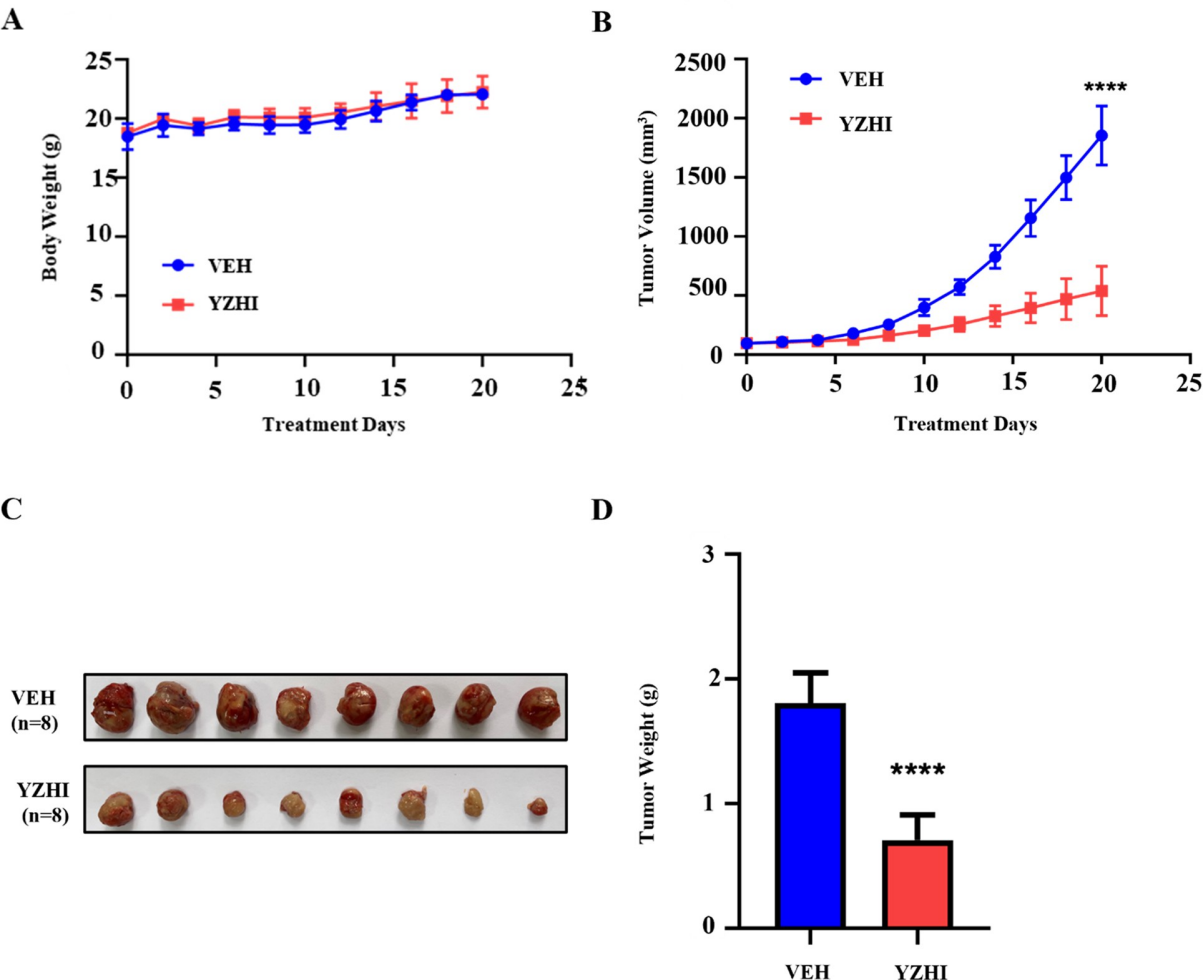
YZHI suppresses xenograft growth in vivo model. (A) The average body weight in YZHI(135.3 mg/kg/d, ip) and vehicle(normal saline, ip) groups. (B) xenograft size from nude mice in YZHI and vehicle groups. (C) xenograft excised from nude mice in each group. (D) The average weight of each xenograft. VEH n = 8, YZHI n = 8. The data were shown as the mean ± SD, **p < 0.01, ****p < 0.0001.

YZHI could inhibit AML cell growth and induce apoptosis in vitro, as shown above, we would explore whether it was still effective in vivo model. HL-60 cells were inoculated subcutaneously into both hind limbs of nude mice, and YZHI was administered at 135.3 mg/kg/d intraperitoneally, which was corresponding to the maximum dose of YZHI injected intravenously in human conversion guideline and the clinic following the animals [25]. There was not a significant difference in body weight between two groups.(Fig 5A and S5A Fig). But we found that YZHI inhibited obviously subcutaneous xenograft growth in nude mice.(Fig 5B–5D, S5A and S5B Fig).

## 4. Discussion

Despite advances in therapeutic strategies, there is still a high resistance to the standard treatment of AML [26]. The characteristic with multiple components and multiple targets of TCM provides an alternative regimen for the treatment of AML. YZHI is a traditional Chinese patent medicine for the treatment of acute and chronic hepatitis. It has been used in the clinic for twenty years, and the overall safety has been approved. In line with this point, we observed that YZHI didn’t obviously affect the proliferation of normal cell lines from Live (Lo2) and prostate stroma (WPMY-1) (Table 1, S1 Table). For the first time, our study reported the anti-cancer activities of YZHI both in vitro and in vivo and provided the rationality for repurposing the drug for AML treatment.

**Fig 6:**
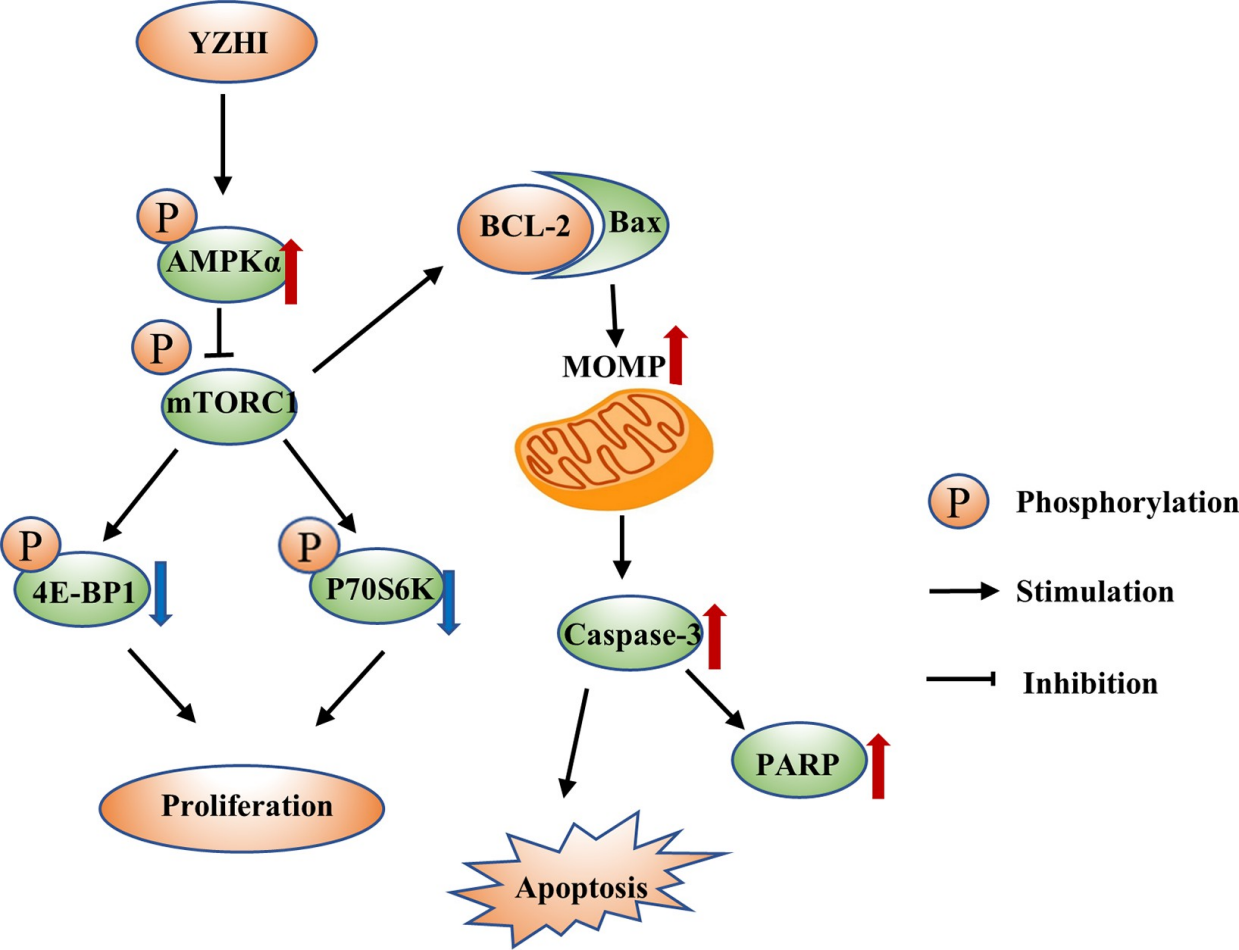
The proposed mechanism of YZHI.

AMPK, which monitors the bioenergetic state, is a highly conserved Ser/Thr protein kinase complex [21]. mTORC1 is one of the major downstream targets negatively regulated by AMPK [27]. The signaling axis plays a critical role in tumorigenesis and thus an attractive target for cancer treatment [28–30]. AMPK activator metformin and 5-aminoimidazole-4-carboxamide ribonucleotide (AICAR) have been shown promising in treating colon cancer, hepatocarcinoma, lymphoma and prostate cancer through inhibition of mTORC1 [31–34]. In this study, we showed that YZHI induced apoptosis and inhibited the proliferation of AML cells by the regulation of the AMPK/mTORC1 signaling pathway (Fig 6). On the other hand, our study may also provide mechanistic insights of YZHI on hepatitis, in which the AMPK/mTORC1 signaling pathway also plays an essential role [35].

The most abundant compound in YZHI is baicalin, which is from Huangqin [36]. In line with our findings, activating the AMPK/mTOR signaling pathway, baicalin suspresses the migration and proliferation of human non-small cell lung carcinoma cells [37]. In addition, YZHI contains quercetin, luteolin, kaempferol, beta-sitosterol, stigmasterol and isorhamnetin, which have been shown to induce tumor cell apoptosis through the AMPK/mTORC1 signaling pathway [38–41]. Future studies are needed to compare these compounds’ effect on the activity of the AMPK/mTORC1 pathway and the anti-cancer efficiency.

In recent decade, some serious adverse drug reaction have raised concerns about the potential toxicity and safety related to TCM injections [42]. Compared with other formulations, TCM injections have advantages of excellent drug-likeness, including good bioavailability and solubility [43]. In addition, TCM injections have been widely used in acute or severe diseases due to the rapid and accurate curative effect. Therefore, we believe that TCM injections provide valuable sources for research and application of drugs. YZHI can be reformulated with better quality control properties and less complexity, or active ingredients can be identified from YZHI to develop new drugs against AML.

## Supporting information

S1 Fig Original data underlying cell viability in Fig 1A–1D. (XLSX)

S2 Fig Original data(A) and blot images(B) underlying cell cycle arrest in Fig 2A–2C.
(ZIP)

S3 Fig Original data(A) and blot images(B) underlying apoptosis in Fig 3A–3H.
(ZIP)

S4 Fig Original data(A) and blot images(B) underlying AMPK/mTORC1 signaling pathway in Fig 4A–4F.
(ZIP)

S5 Fig Original data(A) and xenograft images(B) underlying xenograft growth in Fig 5A–5D.
(ZIP)

S1 Table Original data underlying cell viability in Table 1. (XLSX)
