# Correction: Yinzhihuang injection induces apoptosis and suppresses tumor growth in acute myeloid leukemia cells

**DOI:** 10.1371/journal.pone.0317018

**Published:** 2025-01-02

**Authors:** Zhe Huang, Yunfu Shen, Xianming Fan, Qulian Guo, Wenzhe Ma

There are errors in the Author Contributions. The correct contributions are:

**Conceptualization:** Zhe Huang, Yunfu Shen, Qulian Guo, Wenzhe Ma

**Investigation:** Zhe Huang, Yunfu Shen, Qulian Guo

**Methodology:** Zhe Huang, Yunfu Shen, Qulian Guo

**Supervision:** Wenzhe Ma

**Validation:** Zhe Huang, Yunfu Shen, Qulian Guo

**Writing–original draft:** Zhe Huang, Yunfu Shen

**Writing–review & editing:** Zhe Huang, XianMing Fan, Wenzhe Ma
