# Supplementary figures and images for: Yinzhihuang injection induces apoptosis and suppresses tumor growth in acute myeloid leukemia cells
Source: PLoS One. 2023 Oct 10;18(10):e0289697. doi: 10.1371/journal.pone.0289697 (PMC10564230; doi:10.1371/journal.pone.0289697)

## Slide 1
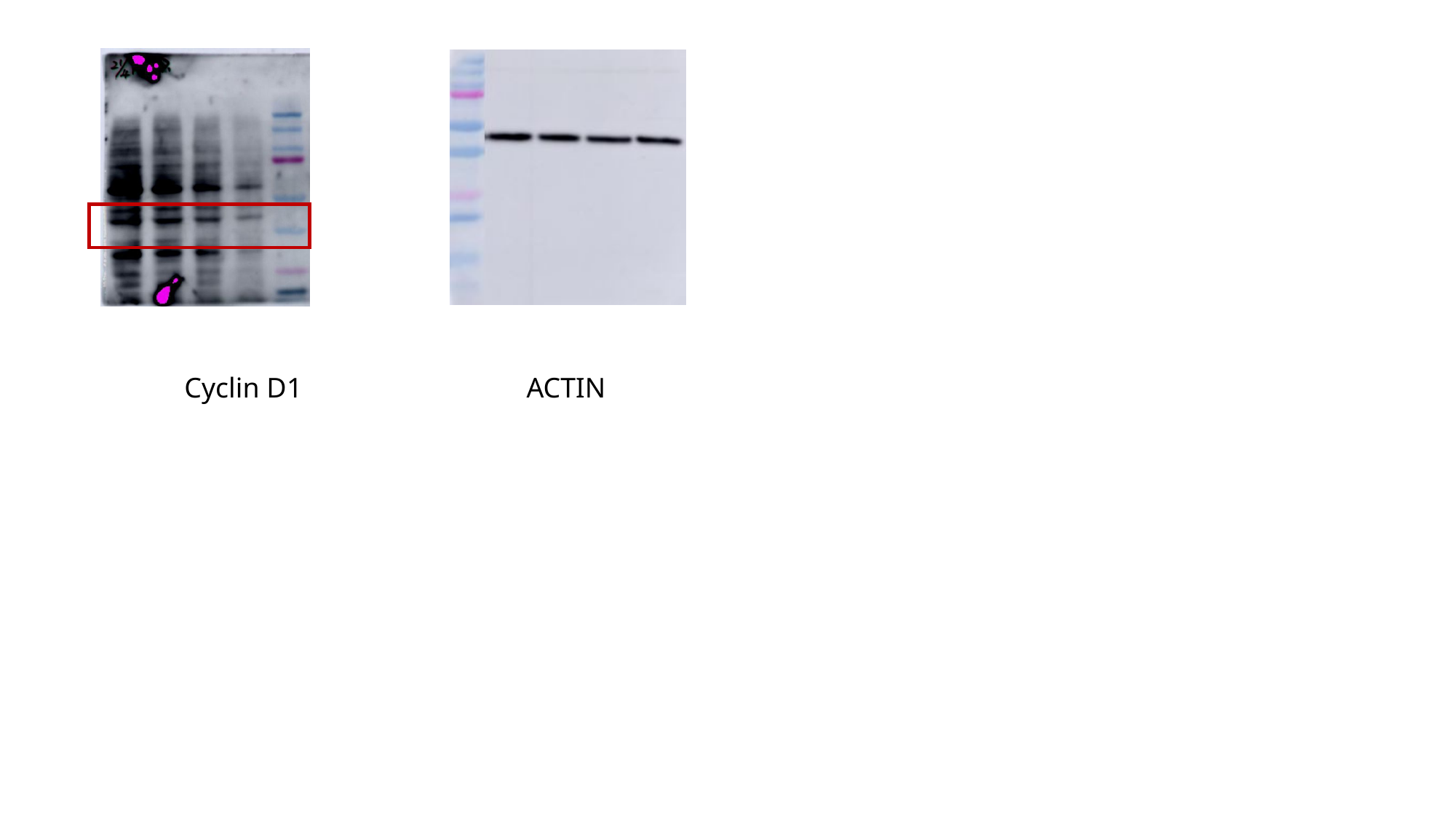

Cyclin D1
ACTIN

Supplement: S2 Fig — Original data(A) and blot images(B) underlying cell cycle arrest in Fig 2A–2C. (ZIP) [file pone.0289697.s002.zip › Fig. S2B.pptx]

## Slide 1
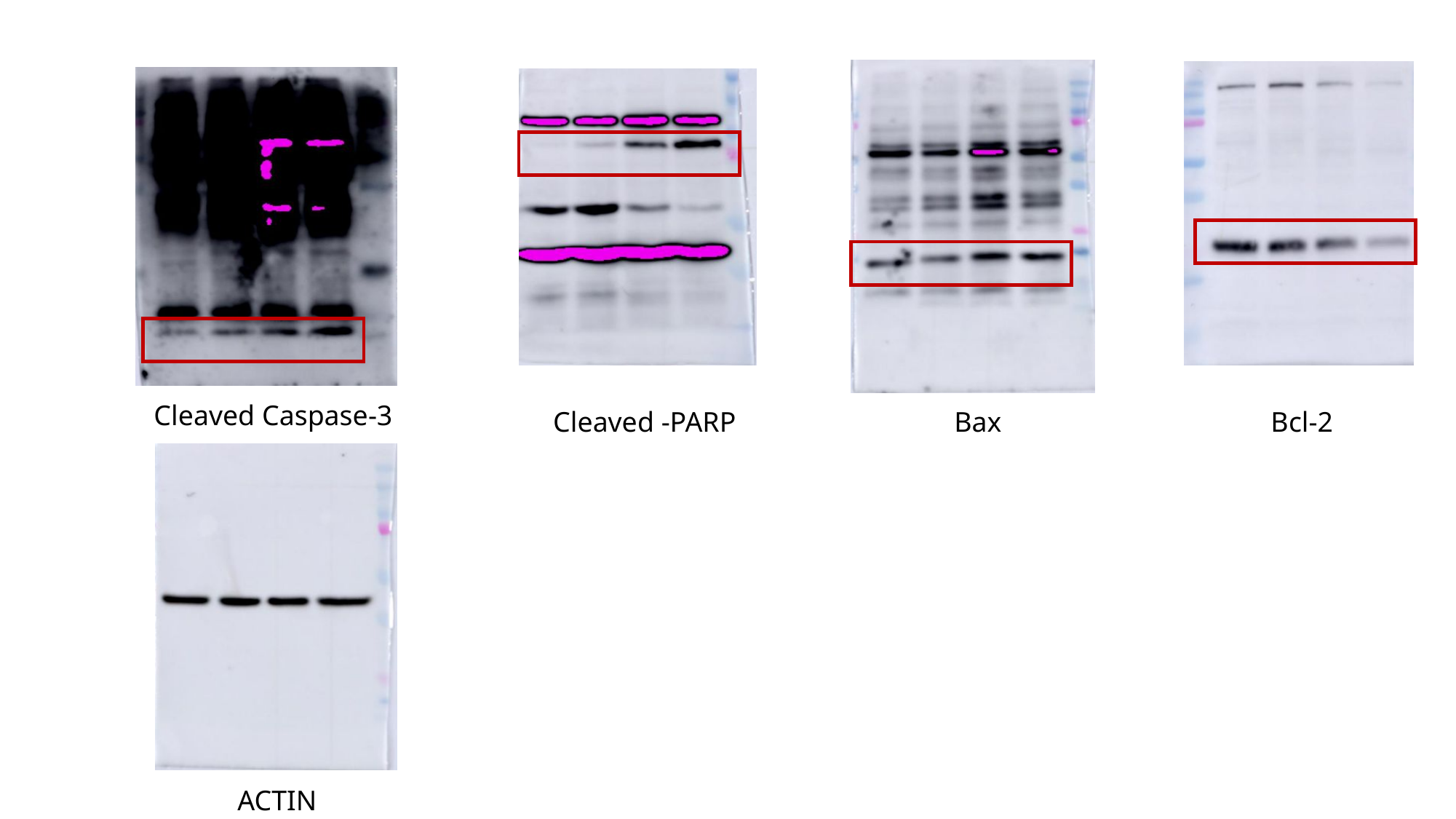

Cleaved Caspase-3
Cleaved -PARP
Bax
Bcl-2
ACTIN

Supplement: S3 Fig — Original data(A) and blot images(B) underlying apoptosis in Fig 3A–3H. (ZIP) [file pone.0289697.s003.zip › Fig. S3B.pptx]

## Slide 1
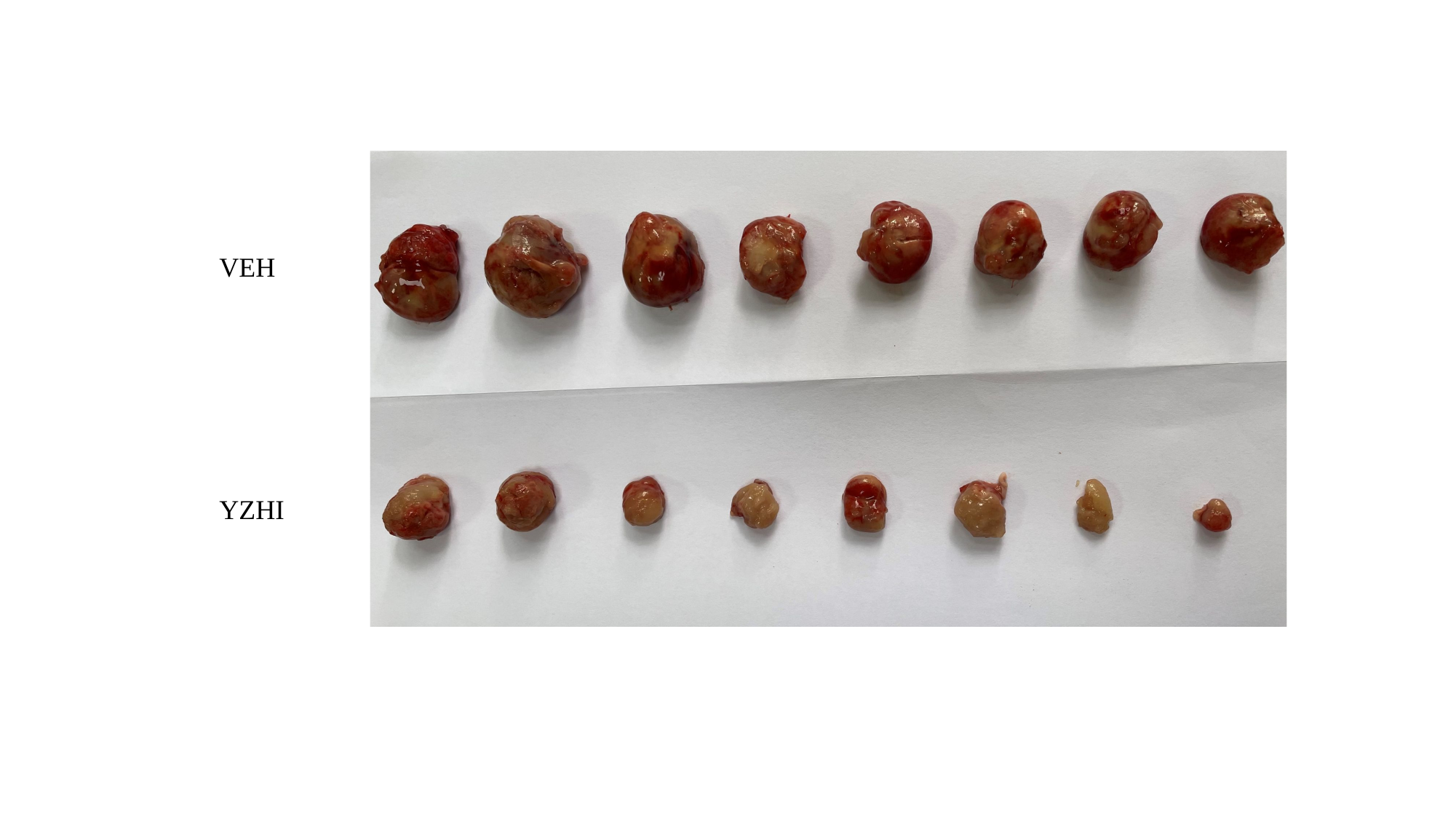

VEH
YZHI

Supplement: S5 Fig — Original data(A) and xenograft images(B) underlying xenograft growth in Fig 5A–5D. (ZIP) [file pone.0289697.s005.zip › Fig. S5B.pptx]
