# Supplementary material for: Yinzhihuang injection induces apoptosis and suppresses tumor growth in acute myeloid leukemia cells
Source: PLoS One. 2023 Oct 10;18(10):e0289697. doi: 10.1371/journal.pone.0289697 (PMC10564230; doi:10.1371/journal.pone.0289697)
Supplement: S4 Fig — Original data(A) and blot images(B) underlying AMPK/mTORC1 signaling pathway in Fig 4A–4F. (ZIP) [file pone.0289697.s004.zip › Fig. S4B.pptx]

## Slide 1
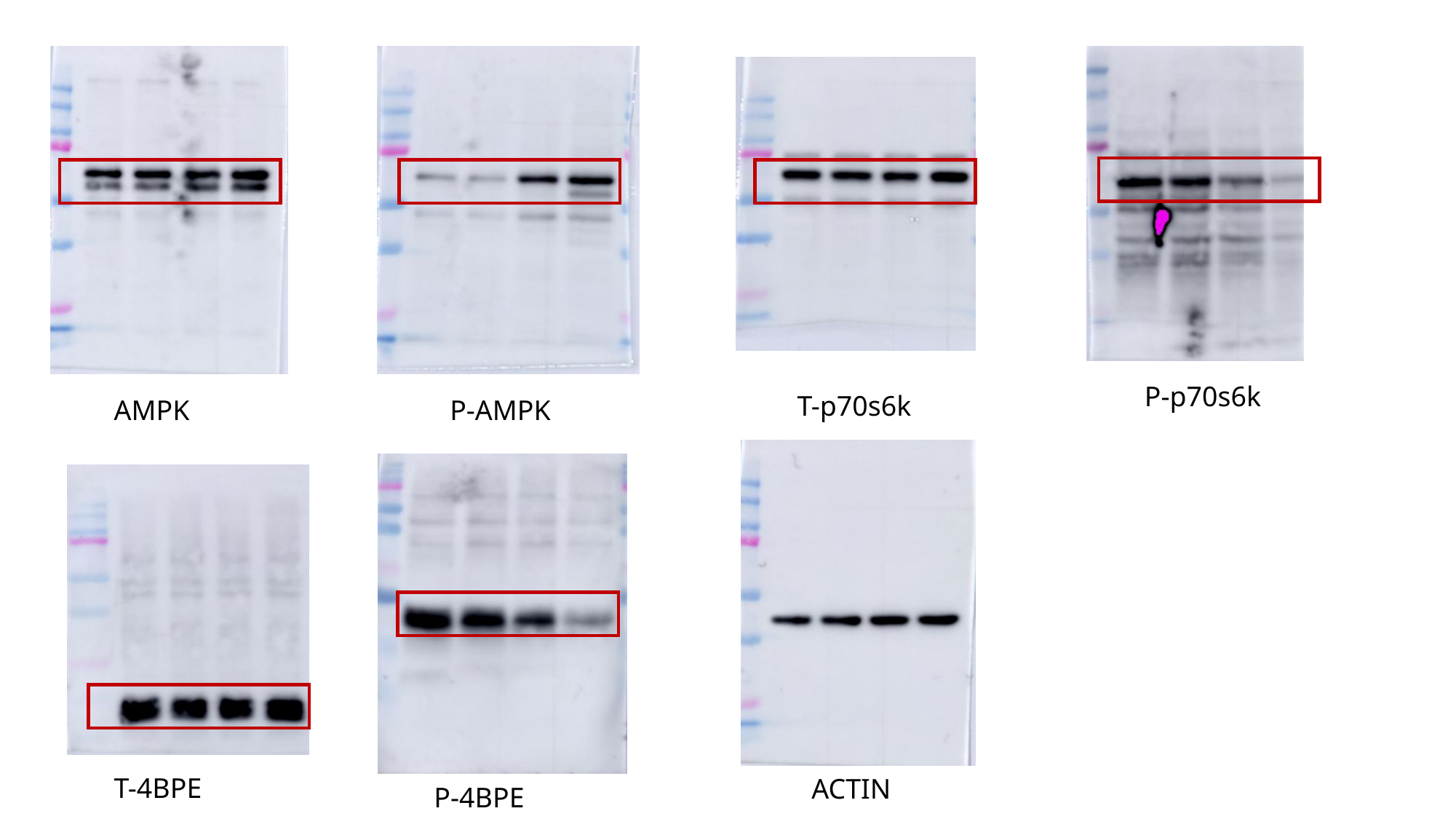

P-p70s6k
T-p70s6k
AMPK
P-AMPK
T-4BPE
ACTIN
P-4BPE

## Slide 2
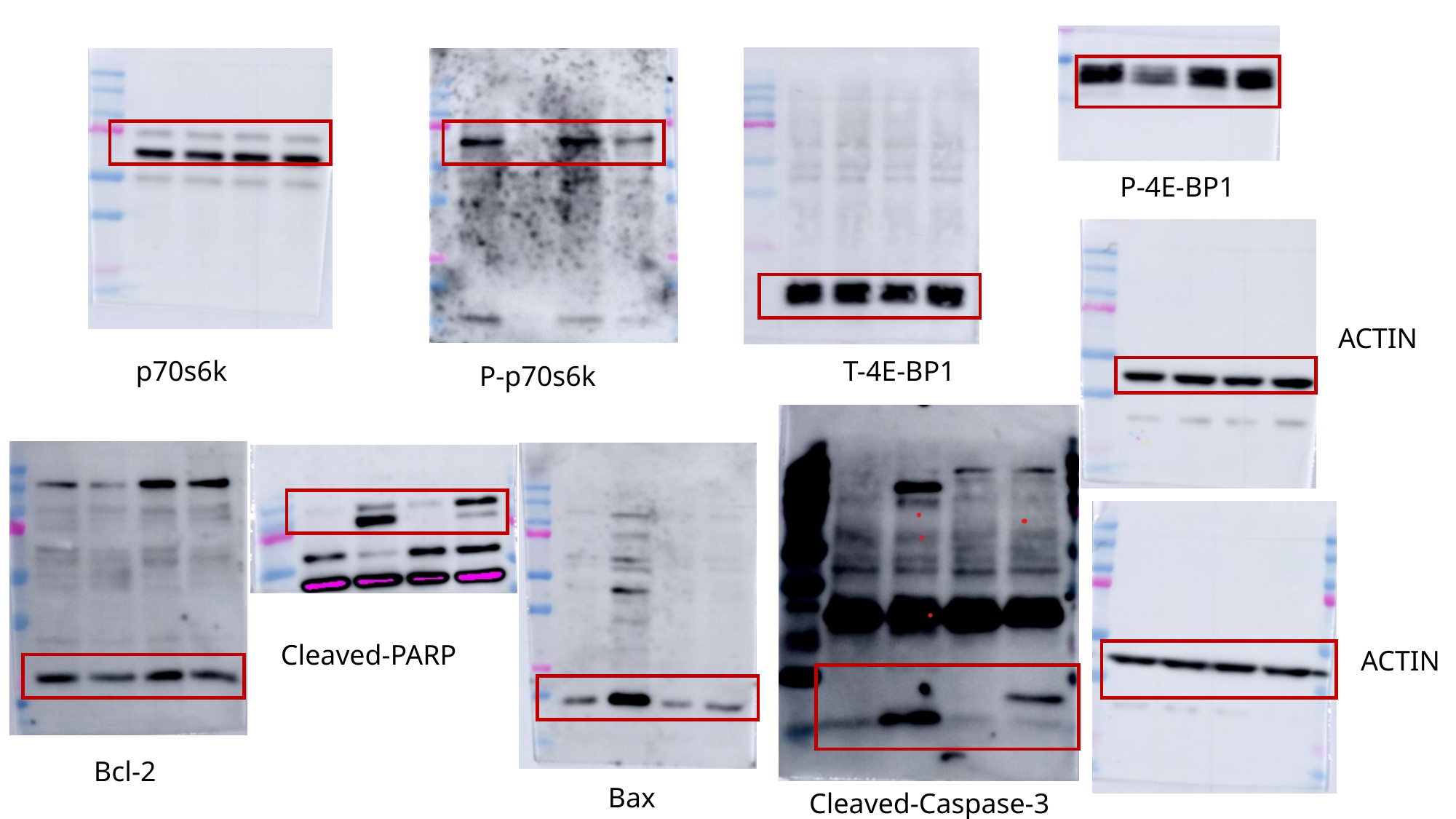

P-4E-BP1
ACTIN
p70s6k
T-4E-BP1
P-p70s6k
Cleaved-PARP
ACTIN
Bcl-2
Bax
Cleaved-Caspase-3
